# Personality Traits and Changes in Health Behaviors and Depressive Symptoms during the COVID-19 Pandemic: A Longitudinal Analysis from Pre-pandemic to Onset and End of the Initial Emergency Conditions in Finland

**DOI:** 10.3390/ijerph18157732

**Published:** 2021-07-21

**Authors:** Tiia Kekäläinen, Enni-Maria Hietavala, Matti Hakamäki, Sarianna Sipilä, Eija K. Laakkonen, Katja Kokko

**Affiliations:** 1 Gerontology Research Center, Faculty of Sport and Health Sciences, University of Jyväskylä, P.O. Box 35, 40014 Jyväskylä, Finland; enni.m.hietavala@jyu.fi (E.-M.H.); sarianna.sipila@jyu.fi (S.S.); eija.k.laakkonen@jyu.fi (E.K.L.); katja.r.kokko@jyu.fi (K.K.); 2 LIKES Research Centre for Physical Activity and Health, Rautpohjankatu 8, 40700 Jyväskylä, Finland; Matti.Hakamaki@likes.fi

**Keywords:** COVID-19, lifestyle, mental health, exercise, eating, alcohol, personality

## Abstract

The COVID-19 pandemic and social distancing measures targeting the transmission of the virus impacted everyday life in 2020. This study investigated pre- to in-pandemic changes in health behaviors and depressive symptoms during the COVID-19 pandemic and the role of personality traits in these changes in Finland. Data from a larger population-based cohort study of 51–59-year-old Finnish women were used (*n* = 358). Self-reported questionnaires gathered information about depressive symptoms, eating behavior, physical activity, and alcohol consumption before the pandemic time, at the onset, and at the end of the COVID-19 emergency conditions. Information about personality traits (extraversion and neuroticism) and sociodemographic factors was available from the pre-pandemic baseline. Women reported more depressive symptoms and unhealthier eating habits at the end of the emergency conditions compared to the pre-pandemic time. An increase in depressive symptoms was associated with changing to unhealthier eating habits. Higher extraversion was associated with a perceived decrease in alcohol consumption and with changing to healthier eating habits. Women with higher neuroticism reported changing to either healthier or unhealthier eating habits. In general, some women reported healthier lifestyle changes while other women reported the opposite. Personality traits help to understand these individual differences in adaptation to the pandemic situation.

## 1. Introduction

The novel coronavirus disease (COVID-19) was discovered at the end of 2019 and rapidly spread all over the world. The World Health Organization announced the pandemic on 11 March 2020, and Europe had become an epidemic epicenter [1]. In order to try to prevent and suppress community transmission, restrictions and recommendations focused on physical distancing [1]. Even though these measures varied between countries from nationwide lockdowns to some social distancing recommendations, the COVID-19 pandemic affected everyday life in all countries. In Finland, in spring 2020, these restrictions included, for example, the closing of schools and most government-run public facilities (e.g., libraries, museums, sports facilities) and a 10-people limit for public gatherings [2]. In addition, people over 70 years of age were recommended to avoid human contact [2].

The working-age population was confronted by many work- and family-related challenges, such as working remotely (from home), being laid off, and homeschooling one’s children during the pandemic. Working-age adults, compared to older adults, reported more COVID-19-related concerns in relation to work goals, finance, and their own emotional well-being; and of the working-age groups, middle-aged adults reported more concerns about other people’s physical health compared to younger and older adults [3]. The pandemic may be challenging particularly to middle-aged adults, who typically have responsibilities for both young and adult children as well as older parents [4]. It is argued that the pandemic has affected especially women by increasing the care burden in families [5].

It is not a surprise that these changes in life have led to a higher mental burden. The COVID-19 pandemic has had a negative impact on mental health and increased the prevalence of depressive symptoms, anxiety, and stress in adult populations [6,7,8,9] especially among women [10,11,12]. More restrictions and the reduction in social contact, as well as higher perceived changes in life, have been associated with increased negative mental health consequences during the pandemic [13]. Good mental health impacts other areas in life and these negative changes in mental health during the pandemic are associated with changes in health behaviors: people who report having suffered because of the pandemic are more likely to report a decrease in physical activity, increase in alcohol consumption, and overeating behavior [10,12,14,15].

Recent research has rapidly focused on the changes in health behaviors occurring during the COVID-19 pandemic. Most studies among adult populations in different countries have found a general decline in physical activity levels during the pandemic compared to the pre-pandemic time [10,12,14,16,17,18,19,20]. However, in all studies, there was also a varied proportion of adults (6–36%) who increased their physical activity [10,12,14,16,17,18,19,20], and in some studies, increasing physical activity was more common than its decrease [15,21,22]. Similarly, while most people seemed to maintain their alcohol consumption at the same level as before the pandemic, some studies found more people to have decreased rather than increased it [16,23,24,25] or vice versa [14,15,26,27,28]. In line with alcohol consumption, while most people seemed to maintain their eating habits [29,30], about every third or fourth individual reported healthier eating habits [14,15,23]. Healthier eating habits included a general perceived change toward healthier eating [14,15] and an increase in the consumption of healthy products, such as fruits, vegetables, nuts, and legumes [23]. Some studies reported a general increase in unhealthy eating behaviors, such as unhealthy snacking, emotional eating, and overeating [16,29,30,31]. Studies have shown that especially adults with lower socioeconomic status are at higher risk for pandemic-related negative changes in their health behaviors [15,21]. Additionally, the shift to remote work (home-based) has been associated with an increase in sedentary time [32] and alcohol consumption [24]. The changes in health behaviors seem to accumulate: participants who increased their physical activity were also more likely to eat healthier and decrease alcohol consumption [14,30].

Overall, the recent research indicates that the COVID-19 pandemic period has not affected all individuals equally. So far, very little attention has been paid to the role of personality traits in relation to health behaviors during the pandemic period, even though personality traits are shown to predict pandemic-related behavior, such as sheltering-in-place [33], preparatory behaviors [34], and social distancing [35]. Personality traits describe relatively stable patterns of thinking, feeling, and behaving [36], and they have been shown to drive behavior even in highly controlled situations like when governments take strong actions to control behavior [33]. High scores in neuroticism describe emotional instability and the tendency to have negative feelings, which have been associated with higher perceived stress and poorer mental health during the pandemic [37,38,39,40], and, in one study, with a decrease in physical activity levels during the pandemic [41]. Extraversion indicates a social, outgoing, and active personality, and it is positively associated with increased physical activity during the pandemic [20,39]. Data about the role of personality traits in health behaviors during the pandemic are limited.

In the unexpected pandemic situation, most previous studies have been limited to cross-sectional data collected during the pandemic measuring self-reported change or assessing behavior before the pandemic retrospectively. In addition, research has been mostly restricted to non-representative online samples. The purpose of the present study was to examine pre- to in-pandemic changes in health behaviors and depressive symptoms and to investigate the moderator role of personality in these changes among middle-aged Finnish women. Longitudinal data utilized in the present study were collected before the COVID-19 pandemic, at the onset (March–April 2020) and at the end (May–June 2020) of the emergency conditions caused by the COVID-19 pandemic and declared by the Finnish government. From now on, the used data collection time points are respectively termed as pre-pandemic, in-pandemic-I, and in-pandemic-II.

## 2. Methods

### 2.1. Participants

The present study uses longitudinal data with three data collection waves—pre-pandemic, in-pandemic-I, and in-pandemic-II—obtained at the study Estrogen, microRNAs, and the risk of metabolic dysfunction (EsmiRs). EsmiRs is a follow-up study of the Estrogenic Regulation of Muscle Apoptosis (ERMA) study. The ERMA study is a population-based cohort study of 47 to 55-year-old Finnish women living in the city of Jyväskylä, Finland, and neighboring municipalities (Kovanen et al., 2018). The consent for future research contacts was obtained from 58% of ERMA participants (*n* = 811), and they were contacted about four years later to participate in the EsmiRs study. The data collection for the EsmiRs study started in November 2018. Prior to 16 March 2020, 445 women had replied to the study invitation and 441 of them filled in the baseline questionnaire, which formed a pre-pandemic datapoint for the present study.

**Figure 1:**
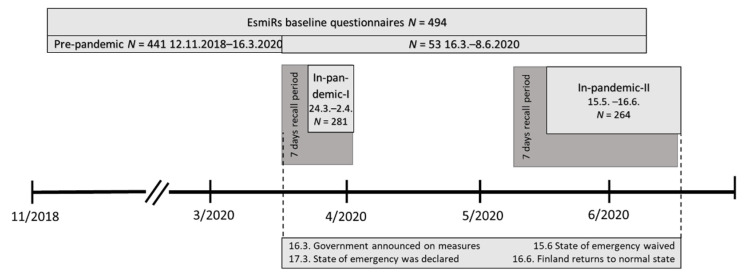
Timelines for the EsmiRs baseline and COVID-19 questionnaires. Dates are day.month.year.

**Figure 2:**
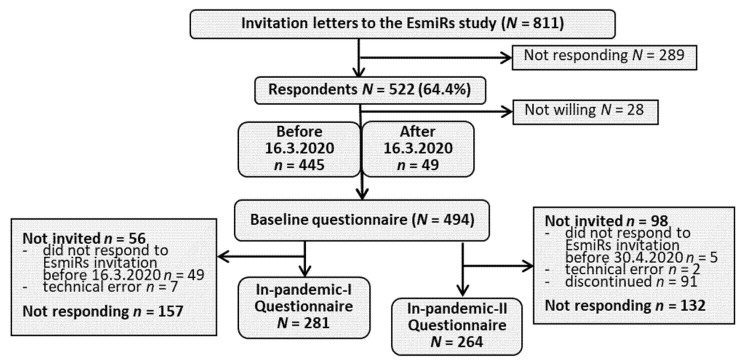
Flow of participants. Figure legend: The flow shows the number of participants who responded to the EsmiRs study invitation before and after the Finnish government announced the emergency conditions. Excluded from the longitudinal analysis of the current study were 49 participants who responded after 16 March 2020 and 4 participants who had filled in the pre-pandemic EsmiRs questionnaire after 16 March 2020.

On 17 March 2020, the Finnish Government submitted decrees concerning the use of powers under the Emergency Powers Act to Parliament because of the SARS-CoV-2 outbreak [2]. In addition, various recommendations and guidelines were issued on restrictions on gatherings, public events, recreational facilities, sports competitions and series, leisure travel, and remote work. The state of emergency was lifted three months later, on 16 June. Even though the state of emergency was lifted on 16 June, various restrictions on gatherings and public events remained in force. The EsmiRs COVID-19 questionnaires were launched at the onset (in-pandemic-I questionnaire) and at the end of emergency conditions (in-pandemic-II questionnaire). The timeline of the questionnaires is shown in Figure 1 and the flow of the participants in Figure 2.

The first COVID-19-related questionnaire (in-pandemic-I) was launched on 23 March 2020 and sent to 438 women who had replied to the EsmiRs study invitation before 16 March (pre-pandemic). The response rate was 64% (*n* = 281) and responses were collected between 24 March–2 April 2020. The second COVID-19 questionnaire (in-pandemic-II) was sent on 14 May 2020 for women who had replied to the EsmiRs baseline questionnaire before 30 April 2020 (*n* = 396). This included 47 women who had replied to the EsmiRs baseline questionnaire between 16 March and 30 April 2020. The response rate for in-pandemic-II was 67% (*n* = 264) and responses were given between 15 May and 16 June 2020. The present study utilizes data from participants who replied to at least one COVID-19-related questionnaire (*n* = 358). Of them, 26% (*n* = 94) replied only to in-pandemic-I, 22% (*n* = 77) only to in-pandemic-II, and 52% (*n* = 187) to both.

### 2.2. Measures

Changes in health behaviors were assessed according to (1) perceived change asked about during the pandemic period, and (2) calculated change in repeated self-reported measures asked about before and during the pandemic. In the questions regarding the perceived change, participants were asked to compare their current situation during the pandemic to January or February 2020.

Leisure-time physical activity was asked about in the EsmiRs baseline survey, and in-pandemic-I and -II questionnaires, including questions about frequency, mean intensity, and mean duration of leisure-time physical activity, which were recorded as MET (metabolic equivalent of task) hours per day [42]. Perceived change in physical activity was asked about in the in-pandemic-I and -II questionnaires with the question: “Have you changed your physical activity or exercise behavior during the COVID-19 pandemic?”; the response options were: No (=1), Yes, increased a lot (=2); Yes, increased a little (=3); Yes, reduced a little (=4); and Yes, reduced a lot (=5). For further analysis, these were recorded as: Decreased (=−1, responses 4 and 5), No change (=0, response 1), and Increased (=1, responses 2 and 3).

Alcohol consumption was asked about at the EsmiRs baseline and also in the in-pandemic-II questionnaire with questions about average weekly consumption of beer or equivalents, wine or other mild alcohol beverages and strong alcohol, which were calculated as an average weekly amount of consumed alcohol. Perceived change in alcohol consumption was enquired about in the in-pandemic-I and in-pandemic-II questionnaires and coded as Increased (=1), Decreased (=−1), and No change (=0).

Regularity of eating was assessed in the EsmiRs baseline survey, and also in the in-pandemic-I and in-pandemic-II questionnaires with the question: “Which of the following descriptions corresponds best to your everyday eating?” The response options were: I eat regularly: my meal and snack times are fairly constant (=1), I eat quite regularly (=2), I eat quite irregularly (=3), I eat very irregularly: a whole day or days can pass without me eating hardly anything, and then on some days I may eat the amount of food of several normal days (=4). For further analyses, categories 3 and 4 were merged, and the scale was reverse coded so that higher values represented better-eating regularity. Perceived change in eating habits was asked about in the in-pandemic-I questionnaire with multiple questions addressing changes in the consumption of various foods (e.g., “Compared to prior, did you eat vegetables… ?”). Participants were asked to compare their most recent seven days to February 2020, choosing from response options: More (=1), Less (=2), or the Same amount as earlier (=3). Five questions assessed the consumption of healthy food products (vegetables, berries and fruits, fish, whole grains, and low-fat dairy products, and seven questions concerned unhealthy products (processed meat, convenience or fast food, refined grains or sugar products, high-fat dairy products, juice or soft drinks sweetened with sugar, sweets, and snacks). The classification of healthy and unhealthy food products is based on Finnish nutrition recommendations [43]. Response options were recoded as: More (=1), Less (=−1), and Same as earlier (=0), and; the mean value of the five questions for healthy products and seven questions for unhealthy products were calculated separately. In the in-pandemic-II questionnaire, self-reported change was asked about with a single question: “Do you think your eating habits have become… ?”; and the response options were coded as Unhealthier (=−1), No change (=0), Healthier (=1).

Depressive symptoms were measured by the Centre for Epidemiological Studies-Depression Scale (CES-D) [44] in the EsmiRs baseline survey and the in-pandemic-II questionnaire. Participants’ experiences and feelings during their past week were enquired about with 20 items (e.g., “I felt lonely” and “I was bothered by things that usually do not bother me”; five were reverse items (e.g., “I felt hopeful about the future”). The response scale was: Seldom or never (=0), Sometimes (=1), Rather often (=2), and Almost all the time (=3). After reverse coding of the reverse items, the sum score was calculated. Cronbach’s alphas for the scale were 0.89 for the pre-pandemic and 0.90 for the in-pandemic-II questionnaire.

Personality traits of extraversion and neuroticism were assessed using the modified short form of the Eysenck Personality Inventory [45] in the EsmiRs baseline survey. The inventory includes nine items for extraversion (e.g., “Are you lively and talkative?”) and ten for neuroticism (e.g., “Are you extremely sensitive in some situations?”), with responses provided in binary form: No (=0) and Yes (=1). Extraversion and neuroticism scales were formed by computing a sum of the scale items. Cronbach’s alphas were 0.80 for extraversion and 0.66 for neuroticism for the pre-pandemic sample. Deleting any of the neuroticism items would not have improved the reliability.

Background variables: Work situation and changes in it were enquired about in the in-pandemic-I and -II questionnaires with several questions asking about participants’ work situation and type of workplace before the pandemic and during the last seven days. Participants’ change in their work situation in the in-pandemic-I and -II was categorized as either: No change (=0, includes participants who were not working before the pandemic, or who continued to work outside the home, or who worked at least partly from home before the pandemic, or who were having a day off, or were on holiday or sick leave during their past seven days) or Change (=1, shifted to working remotely at home, reported an increase in remote work from home, or were laid off or made redundant). Education, work situation, and marital status were asked about in the baseline questionnaire. The participants’ education range was categorized into two groups: those with a lower or upper secondary school education or at most a vocational degree (=0), and those with tertiary education, such as a Bachelor’s or higher university degree (=1). Participants’ work situation was categorized as: Not employed (=0, including parental leave, sickness or disability pension, unemployed, or student), or Employed (=1). Marital status was categorized as: Not living with a partner (=0, including being single, divorced, separated, widowed, or in a permanent relationship but not living together), or Living with a partner (=1, including being married or cohabiting).

All participants who replied to questionnaires pre-pandemic (*n* = 358), at the beginning of emergency conditions (in-pandemic-I, *n* = 281), and at the end of emergency conditions (in-pandemic-II, *n* = 264) replied to all questions with the exception of personality traits and depressive symptoms at the baseline (1 missing values for each).

### 2.3. Statistical Analyses

Statistical analyses were performed using IBM SPSS Statistics 24.0. Means, standard deviations, and frequencies were used for descriptive purposes. Differences in repeated variables were analyzed with a paired samples *t*-test for continuous variables (depressive symptoms, alcohol consumption, and leisure-time physical activity MET-hours), with the Wilcoxon signed-rank test for ordinal variables (eating regularity, alcohol consumption, and physical activity). Associations between changes in different variables were analyzed with Pearson and Spearman correlations. Difference scores for repeated variables were calculated for correlation analyses by subtracting the pre-pandemic value from the in-pandemic-I or -II value.

A generalized estimating equation (GEE) approach with an unstructured working correlation matrix and a linear model was used to analyze changes in study variables measured at two or three time points. The GEE model utilizes information also from incomplete observations (e.g., data on participants whose information is available from the pre-pandemic and in-pandemic-I questionnaires but not from the in-pandemic-II questionnaire) and takes into account correlations between outcomes across time for the same participant. For these analyses, only participants who replied to the baseline questionnaire before 16 March 2020 were included. Because the timing of the baseline questionnaire varied between November 2018 and March 2020, the time gap between these was calculated. First, we analyzed the effect of time on outcomes and included time and control variables (the time gap, education, marital status, and change in work status). Second, we analyzed the moderator role of personality traits by including personality traits and interaction terms between personality traits and time to the models. Wald chi-square test values and *p*-values were reported. For interaction figures, extraversion was divided into tertiles based on its distribution.

Regression analyses were performed to analyze the associations of personality traits with perceived change in health behaviors. Linear regression analyses were used for continuous-type outcomes (perceived changes in eating healthy or unhealthy products in the in-pandemic-I questionnaire), ordinal regression for ordinal outcomes (perceived changes in physical activity in the in-pandemic-I questionnaire, and alcohol consumption in the in-pandemic-I and -II questionnaires) and multinomial regression for ordinal outcomes that did not meet the assumption of proportional odds (i.e., statistically significant test of parallel lines) (perceived changes in physical activity and eating behavior in the in-pandemic-II questionnaire). Standardized beta coefficients (β) with *p*-values were reported for linear regression analyses and odds ratios with a 95% confidence interval for ordinal and multinomial regression analyses.

## 3. Results

### 3.1. Descriptive Statistics

The comparison between the study’s sample and the Finnish population of the same age, as well as between the sample of participants in the present study and non-participants, is shown in the supplementary material (Tables S1 and S2). Briefly, non-smoking women with higher education and normal body mass index (BMI) were overrepresented in the present study compared to the Finnish population samples of the same age. Participants in the present study (*n* = 358) were younger (*M* = 54.33, *SD* = 2.04 vs. *M* = 54.99, *SD* = 2.06; *p* = 0.002) and had a higher education (47% vs. 34% with tertiary education; *p* = 0.010) at the EsmiRs baseline compared to participants who did not respond to either of the in-pandemic questionnaires and were subsequently not analyzed in the present study (*n* = 136). Further, they did not differ in marital status, work situation, or any other study variables (depressive symptoms, extraversion, neuroticism, weekly alcohol consumption, regularity of eating, and leisure-time physical activity MET-hours) at the baseline.

Descriptive statistics at the pre-pandemic time are shown in Table 1. Scores or proportions in study variables at the three data collection waves (pre-pandemic, in-pandemic-I, and in-pandemic-II) are shown in Table 2.

Participants had more depressive symptoms at the end of the emergency conditions (in-pandemic-II) compared to the pre-pandemic time (paired samples *t*-test: *t* = −3.17, *df* = 262; *p* = 0.002). In addition, participants reported less regular eating at the end of the emergency conditions (in-pandemic-II) compared to the pre-pandemic time (Wilcoxon signed-rank test: *Z* = −2.96; *p* = 0.003) or the beginning of the emergency conditions (in-pandemic-I: *Z* = −3.35; *p* = 0.001). There were no differences in leisure-time physical activity or alcohol consumption between the three measurement points, nor in regard to changes in alcohol consumption or physical activity between the beginning (in-pandemic-I) and end (in-pandemic-II) of the emergency conditions.

### 3.2. Associations between Changes in Health Behaviors and Depressive Symptoms

Correlations between changes in health behaviors and depressive symptoms are shown in the Supplementary Materials (Tables S3 and S4). At the onset of the emergency conditions (Table S3), perceived changes in physical activity correlated with the calculated change in physical activity (r = 0.20, *p* < 0.001), perceived change in alcohol consumption with perceived change in eating healthy (r = 0.14, *p* = 0.022) and unhealthy products (r = 0.25, *p* < 0.001), and perceived change in eating unhealthy products with the calculated change in eating regularity (r = −18, *p* = 0.003).

At the end of emergency conditions (Table S4), perceived changes in physical activity (r = 0.32, *p* < 0.001), eating habits (r = 0.13, *p* = 0.030), and alcohol consumption (r = 0.32, *p* < 0.001), correlated with the corresponding repeated variables change scores calculated between the pre-pandemic time and in-pandemic-II questionnaire. Perceived change in physical activity was positively associated with the perceived change in eating habits (r = 0.29, *p* < 0.001), that is, those who reported having increased their physical activity also reported healthier changes in eating habits. Change score of eating regularity was negatively correlated with the change score of alcohol consumption (r = −0.14, *p* = 0.023), i.e., an increase in eating regularity was associated with a decrease in alcohol consumption. In addition, both perceived change in eating habits (r = 0.21, *p* < 0.001) and change score of eating regularity (r = 0.17, *p* = 0.006), were negatively associated with a change in depressive symptoms, that is, unhealthier changes in eating habits were associated with an increase in depressive symptoms.

### 3.3. Changes in Health Behaviors and Depressive Symptoms and the Role of Personality

Longitudinal changes in repeated measures of health behaviors and depressive symptoms were analyzed by the GEE. The effects of time on outcomes after controlling for the time gap between the pre-pandemic questionnaire and emergency conditions, education, marital status, and change in work are shown in the results in Table 3 in Model 1. There was a statistically significant increase in depressive symptoms from the pre-pandemic time to the end of the emergency conditions and eating regularity decreased from the pre-pandemic time to the end of the emergency conditions.

**Figure 3:**
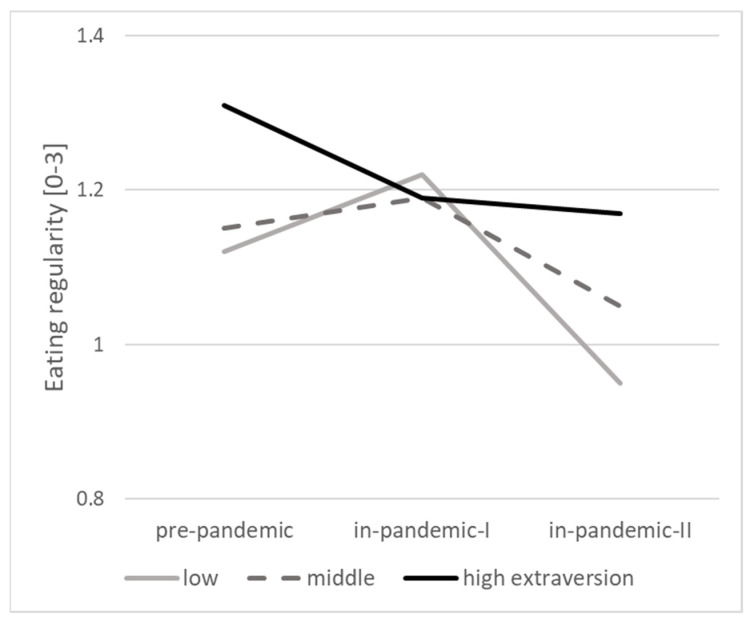
The interaction effect of time and extraversion on eating regularity (*p* < 0.05).

The moderator role of personality on changes is shown in Table 3 in Model 2. Scoring higher in extraversion and lower in neuroticism were associated with lower levels of depressive symptoms and a higher level of leisure-time physical activity. Higher scores in neuroticism were associated with lower eating regularity. There was a statistically significant interaction between extraversion and time for eating regularity, i.e., people scoring low in extraversion were more likely to decrease their eating regularity during the pandemic (see Figure 3).

Associations of personality traits with perceived change in health behaviors are shown in Table 4 for the onset of the emergency conditions (in-pandemic-I) and in Table 5 for the end of the emergency conditions (in-pandemic-I). At the onset of emergency conditions, personality traits were not related to a perceived change in health behaviors. At the end of the emergency conditions, women who scored higher in extraversion were more likely to report a decrease in their alcohol consumption. In addition, women with higher scores in neuroticism were more likely to report a change in their eating behavior, i.e., they reported either a change to healthier or unhealthier eating habits compared to women with lower neuroticism. Women scoring high in extraversion were more likely to report a healthier change in their eating habits. There were no differences between decrease and increase (physical activity) or unhealthier and healthier (eating habits) groups in the multinomial regression analyses.

## 4. Discussion

The purpose of this study was to examine pre- to in-pandemic changes in health behaviors and depressive symptoms as well as the moderator role of personality in these changes in middle-aged women. The results show unhealthier changes in eating habits and an increase in the regularity of depressive symptoms. Changes in eating habits were associated with other changes, i.e., participants who changed their eating habits in an unhealthier direction were more likely to report an increase in depressive symptoms and alcohol consumption as well as a decrease in physical activity. Higher extraversion predicted healthier changes in alcohol consumption and eating habits, while higher neuroticism predicted either healthier or unhealthier changes in eating habits. In general, all changes and associations were not yet seen at the onset of the emergency conditions but occurred by the end of those conditions.

Previous studies have focused on prevalence and change in depressive symptoms during the pandemic without available information on depressive symptoms from the pre-pandemic time [9]. Our finding of an increase in depressive symptoms from the pre-pandemic time to the end of the emergency conditions supports previous studies showing relatively high rates of depressive symptoms during the pandemic [6,9,10,11,12]. In general, the level of depressive symptoms seems to have been highest at the beginning of the restrictions and then people adapted to the circumstances [46,47]. We did not have information about depressive symptoms at the beginning of the emergency conditions, but the negative change in depressive symptoms was seen after two or three months of the pandemic restrictions. Lower extraversion and higher neuroticism were associated with a higher level of depressive symptoms. These results are in line with previous studies suggesting the role of personality traits [48] and marital status [49,50] in depressive symptoms, as well as studies suggesting that the risk factors for poor mental health are the same during a pandemic as in general [9,46,47].

In line with previous findings [29,30], the majority of participants reported at least quite regular eating and no changes in their eating habits during the pandemic. Interestingly, while most previous studies were conducted with a single questionnaire during the pandemic [16,29,30,31], our results revealed that the change to unhealthier eating habits seems to take some time and occurred at the end of the emergency conditions. Consistent with previous studies [14,15,30], unhealthier changes in eating were associated with increased depressive symptoms and alcohol consumption as well as decreased physical activity. It is not possible to draw conclusions regarding causal directions based on this study, but it can be speculated that some women responded to the increased mental burden with unhealthier eating while other women took the opportunity to improve their lifestyle.

It is interesting to note that personality played a role in eating behavior. Consistently, in combination with a perceived change in eating habits and repeated-measures change in eating regularity, lower extraversion predicted unhealthier changes. There is some evidence that extraversion is positively associated with eating more vegetables [51], and negatively associated with emotional eating [52], and it is possible that women scoring high in extraversion have taken the opportunity to make healthier choices in eating behavior during the pandemic. In addition, as people scoring high in extraversion are social and outgoing [36], it is likely that the changes in eating behavior are partly explained by the elimination of social eating practices due to the pandemic. Surprisingly, women scoring higher in neuroticism were more likely to report either unhealthier or healthier changes in their eating habits during the pandemic. These results may tell us something about the two sides of neuroticism [53]. High scores in neuroticism could manifest depressive and anxious mood that is associated with unhealthier eating habits, such as emotional eating [52]. High scores in neuroticism could also lead to vigilant health behavior [53], which is seen, for example, in better compliance with social distancing and hygiene recommendations during the pandemic [54].

In line with other studies [10,12,14,16,17,18,19,20], more women reported a decrease than an increase or no change in their physical activity at the beginning and at the end of the emergency conditions. There was no statistically significant change in the mean level of leisure-time physical activity from the pre-pandemic time to the end of the emergency conditions. Perceived change may tell us more about general changes in all physical activities, including work-related physical activity, commuting, and habitual physical activity, whereas repeated-measures physical activity tells us more about leisure-time exercise behavior. It was previously reported with this same sample that over half of the women reported a change in the type of their physical activity, e.g., a shift from group exercises to outdoor activities [55].

Alcohol consumption showed the highest stability during the pandemic, as about 80% of women did not report a change in their alcohol consumption, and weekly alcohol consumption did not change from the pre-pandemic time onward. Previously published studies evaluating alcohol consumption during the pandemic observed inconsistent results showing both a general increase [14,15,26,28] and a decrease [16,23,24,25] in alcohol consumption, which is likely to be explained by different samples and patterns of alcohol consumption. Women with higher extraversion as well as with higher education were more likely to report a decrease in their alcohol consumption. A possible explanation for these results is that women with both higher extraversion and higher education typically drink alcohol in social situations outside the home and, due to the pandemic, these situations were eliminated.

An interesting finding was that all changes were not seen until the end of the emergency conditions, and the role of personality traits on health behavior change seemed to occur at the same time. As the questions in our in-pandemic-I questionnaire were related to the first few weeks of the emergency conditions in Finland, it is possible that individual differences had less of an impact at the beginning of the unexpected new situation, and that during the next couple of months, individual differences in adaptation were taking shape. In addition, it is important to bear in mind the seasonal variation from late winter to early summer during these emergency conditions.

The generalizability of these results is subject to certain limitations. For instance, as the pandemic situation and pandemic-related restrictions varied between and even within countries, these results represent the situation in central Finland during the spring of 2020. In addition, the sample was highly educated and had a relatively healthy lifestyle compared to the Finnish population of the same age. This is a common bias factor in COVID-19 related studies, since education may explain the willingness to reply to online surveys. People with lower education may be at higher risk for unhealthier lifestyle changes and mental health problems during the pandemic [21,46,56], and, therefore, our results may underestimate the impact of the pandemic among middle-aged women.

The present study contributes to the rapidly expanding field of COVID-19 pandemic research and has some major strengths compared to most other studies. First, we collected information about depressive symptoms and health behaviors earlier in the pre-pandemic time and could examine changes between the pre-pandemic and pandemic times without problems associated with a retrospective inquiry [57]. Second, we also gathered information on perceived changes reported during the pandemic and could consequently see a broader picture of pandemic-related changes with information about both self-rated and repeated-measures changes. Third, the in-pandemic questionnaires were timed to capture the situation right at the onset and again at the end of the emergency conditions, the effective timing of which adds clarity to the findings.

## 5. Conclusions

These results suggest that the COVID-19 pandemic contributed negatively to mental health and to eating habits among middle-aged women. Even though most women reported a decrease in their physical activity, the average level of leisure-time physical activity actually did not change during the pandemic. In general, some people seemed to approach the pandemic situation as an opportunity for healthier lifestyle choices, whereas the opposite was the case for some other people. Personality traits may explain these individual differences in adaptation to the pandemic situation.

## Figures and Tables

**Table 1:** Descriptive statistics at pre-pandemic time (*n* = 358) and in-pandemic change in work (*n* = 281/264).

	Pre-Pandemic		In-Pandemic-I		In-Pandemic-II	
	*M*	*SD*	*n*	%	*n*	%
Age	54.3	2.0				
Extraversion	5.4	2.7				
Neuroticism	2.7	2.1				
	*n*	%				
Education						
Lower or higher secondary	191	53.4				
Tertiary or higher degree	167	46.6				
Marital status						
Living with a partner	266	74.3				
Not living with a partner	92	25.7				
Work situation						
Employed	332	92.7				
Not employed	26	7.3				
Changes in work						
No			197	70.1	160	60.6
Yes			84	29.9	104	39.4

**Table 2:** Study outcomes at three data collection waves.

	Pre-Pandemic *n* = 358		In-Pandemic-I *n* = 281		In-Pandemic-II *n* = 264	
	*M*	*SD*	*M*	*SD*	*M*	*SD*
CES-D	0.45	0.36	n/a		0.53 *^,a^	0.38
Leisure-time physical activity, MET h/d	3.93	3.52	4.16	3.56	4.28	3.56
Weekly amount of alcohol consumption	3.22	3.51	n/a		2.98	3.60
Perceived changes in eating healthy products [−1–1]	n/a		−0.02	0.23	n/a	
Perceived changes in eating unhealthy products [−1–1]	n/a		−0.06	0.27	n/a	
	*n*	%	*n*	%	*n*	%
Regularity of eating, %						
Irregular	34	9.5	21	7.5	39 *^,a,b^	14.8
Quite regular	230	64.2	181	64.4	174	65.9
Regular	94	26.3	79	28.1	51	19.3
Perceived changes in physical activity						
Less			127	45.5	97	42.2
No change			84	30.1	74	32.2
More			68	24.4	59	25.7
Perceived changes in alcohol consumption						
Less			39	14.0	35	13.3
No change			223	79.9	214	81.1
More			17	6.1	15	5.7
Perceived changes in eating habits						
Unhealthier			n/a	n/a	57 *^,b^	21.6
No change					176	66.7
Healthier					31	11.7

* Statistically significant difference (*p* < 0.05) in paired sample analysis compared to ^a^ pre-pandemic time or ^b^ in-pandemic-I questionnaire. n/a = Not available: CES-D and alcohol consumption were not asked in the in-pandemic-I questionnaire, perceived changes in eating habits were asked with different questionnaires in the in-pandemic-I and -II questionnaires.

**Table 3:** Changes in depressive symptoms and health behaviors analyzed by general equation estimation (*n* = 230–441).

	Depressive Symptoms		Alcohol Consumption		Leisure Time Physical Activity		Eating Regularity	
	Wald Test	*p*	Wald Test	*p*	Wald Test	*p*	Wald Test	*p*
Model 1 ^a^								
Time	9.26	<0.001	1.87	0.171	0.95	0.329	14.47	<0.001
Model 2 ^a^								
Time	0.83	0.363	0.18	0.670	0.37	0.545	8.77	0.003
Extraversion	4.67	0.031	0.34	0.560	4.65	0.031	0.34	0.560
Neuroticism	166.10	<0.001	0.29	0.592	4.81	0.028	26.10	<0.001
Extraversion*Time	0.468	0.494	0.32	0.574	1.18	0.555	7.16	0.028
Neuroticism*Time	0.885	0.347	1.21	0.271	0.16	0.921	0.08	0.960

^a^ Models adjusted for the time gap between filling in the pre-pandemic baseline questionnaire and the onset of the emergency conditions, education, marital status, and changes in work during the pandemic.

**Table 4:** Associations of personality traits with perceived changes in health behaviors at the onset of emergency conditions (*n* = 281).

	Alcohol ^a^		Physical Activity ^a^		Eating Healthy Products ^b^		Eating Unhealthy Products ^b^	
	OR	95% CI	OR	95% CI	β	*p*	β	*p*
Extraversion	1.02	0.91–1.15	1.01	0.92–1.10	−0.05	0.439	0.01	0.832
Neuroticism	1.11	0.95–1.30	0.92	0.82–1.04	0.06	0.354	−0.03	0.667

Analyzed with ^a^ ordinal regression analysis, ^b^ linear regression analysis. Models adjusted for education, marital status, and change in work.

**Table 5:** Associations of personality traits with perceived changes in health behaviors at the end of emergency conditions (*n* = 264).

	Alcohol ^a^		Physical Activity				Eating Habits			
			Decrease vs. No Change ^b^		Increase vs. No Change ^b^		Unhealthier vs. No Change ^b^		Healthier vs. No Change ^b^	
	OR	95% CI	OR	95% CI	OR	95% CI	OR	95% CI	OR	95% CI
Extraversion	0.78	0.69–0.92	0.99	0.87–1.13	1.14	0.98–1.33	1.11	0.96–1.28	1.24	1.01–1.52
Neuroticism	0.92	0.78–1.10	1.12	0.95–1.34	1.14	0.94–1.39	1.26	1.05–1.51	1.51	1.18–1.93

Analyzed with ^a^ ordinal regression analysis, ^b^ multinomial regression analysis. Models adjusted for education, marital status, and change in work.

## Data Availability

The data presented in this study are available on request from Dr. Eija Laakkonen (eija.k.laakkonen@jyu.fi). The data are not publicly available due to ethical and privacy restrictions.

## Supplementary Materials

The following are available online at https://www.mdpi.com/article/10.3390/ijerph18157732/s1. 1: Representativeness of the sample (Tables S1 and S2). 2: Associations between changes in health behaviors and depressive symptoms (Tables S3 and S4).
