# Supplementary material for: Personality Traits and Changes in Health Behaviors and Depressive Symptoms during the COVID-19 Pandemic: A Longitudinal Analysis from Pre-pandemic to Onset and End of the Initial Emergency Conditions in Finland
Source: Int J Environ Res Public Health. 2021 Jul 21;18(15):7732. doi: 10.3390/ijerph18157732 (PMC8345535; doi:10.3390/ijerph18157732)
Supplement: Supplementary file 1 [file ijerph-18-07732-s001.zip › ijerph-1301202-supplementary.pdf]

Supplementary Material 1: Representativeness of the sample

Comparison to the Finnish population

Comparisons between the present study sample (*n* = 358), the EsmiRs (Estrogen, MicroRNAs and the Risk of Metabolic Dysfunction) study sample (*n* = 494), the ERMA (Estrogenic Regulation of Muscle Apoptosis) study sample (*n* = 1098), and the Finnish population regarding education, marital status and lifestyle factors are shown in Table S1.

The ERMA baseline sample consisted of women aged 47 to 55 years, and the EsmiRs study sample, as well as the sample used in the present study, consisted of women aged 51 to 59 years who had participated in the ERMA baseline study around four years before (2015-2016). The sample of the present study included women who replied to at least one COVID questionnaire in the spring of 2020, while the EsmiRs study sample included an additional 136 women who replied to the EsmiRs baseline questionnaire but not to the COVID questionnaires.

The ERMA baseline sample can be considered to be representative of the healthy, non-obese middle-aged female population in Finland [1]. Women who responded to the pre-study questionnaire but did not continue to the baseline measurements had in more cases undergone an oophorectomy and were less likely to use hormonal contraception than those who participated in the baseline measures [1]. This is not likely to affect the generalizability of the results.

The information about education, marital status and lifestyle factors in the Finnish middle-aged female population was used to compare the representativeness of the sample. Information on education and marital status of Finnish women aged 45 to 59 years was available from the Official Statistics of Finland database [2,3]. Information regarding alcohol consumption, smoking, body mass index (BMI), and accelerometer-based moderate to vigorous daily physical activity (MVPA) was available from the latest population-based studies [4–7]. Comparisons between the samples regarding education, proportion of smokers, and normal weight participants (BMI <25) were analyzed with chi-square tests and adjusted residuals. Statistical comparisons of marital status, alcohol consumption, and MVPA were not

reasonable because different categorization methods (marital status) and different questionnaires (alcohol consumption) were used for the study samples and the Finnish population sample; furthermore, for the MVPA only a rough mean was available for the population sample.

Women with lower education were underrepresented and women with higher education overrepresented in the ERMA and EsmiRs study samples, and the same is true for the sample used in the present study compared to the Finnish female population of the same age (see Table S1). The proportion of married women was slightly higher in all three samples compared to the Finnish population.

The average amount of alcohol consumption and the proportion of alcohol users at risk is relatively similar across the three study samples and the Finnish female population [4,5]. It is worth keeping in mind that the amount of alcohol consumption is likely to be underestimated in studies compared to the whole population, since those with the heaviest alcohol consumption are most likely not to respond to study invitations [4]. In addition to alcohol consumption, the average amount of whole-day accelerometer-based MVPA was relatively similar across the three study samples compared to the Finnish female population [6].

The average BMI seems to have been slightly lower and normal-weight participants overrepresented in the three study samples compared to the Finnish female population sample. This is likely due to the exclusion of severely obese participants (BMI >35) from the ERMA study. The proportion of women aged 50 to 59 years having a BMI higher than 35 is 11.4% in Finland [7]. In addition, daily smokers were underrepresented in the three study samples compared to the Finnish female population of the same age [7].

Comparison between the sample of the present study and nonparticipants

Comparisons between the sample of the present study and nonparticipants regarding characteristics measured at the baseline of the EsmiRs study are presented in Table S2. The participants comprising the present study's sample were younger and more educated than nonparticipants, but they did not differ in any other study variables.

References

1. Kovanen V, Aukee P, Kokko K, Finni T, Tarkka IM, Tammelin T, et al. 2018. Design and protocol of Estrogenic Regulation of Muscle Apoptosis (ERMA) study with 47 to 55-year-old women's cohort: novel results show menopause-related differences in blood count. Menopause 25:1020–32.

2. Official Statistics of Finland. Educational structure of population [e-publication] [Internet]. [cited 2020 Oct 2]. Available from: http://www.stat.fi/til/vkour/index_en.html

3. Official Statistics of Finland. Changes in marital status [e-publication]. [Internet]. [cited 2020 Oct 2]. Available from: http://www.stat.fi/til/ssaaty/index_en.html

4. Härkönen J, Savolainen J, Virtala E & Mäkelä P. 2017. Suomalaisten alkoholinkäyttötavat 1968-2016. Juomatapatutkimusten tuloksia.[Results from the Finnish Drinking Habits Surveys 1968–2016]. Helsinki: Terveyden ja hyvinvoinnin laitos.

5. Lintonen T, Niemelä S & Mäkelä P. 2019. Alkoholinkäytön hälytysrajan ylittäviä käyttäjiä on Suomessa vähintään viisi prosenttia väestöstä [At least five percent of the Finnish population are high risk alcohol users. Results from the Drinking Habits Survey 2016.] Lääketieteellinen aikakauskirja Duodecim 135(16):1459-66.

6. Husu P, Tokola K, Suni J, Vähä-Ypyä H, Mänttäri A, Vasankari T, et al. 2018. Suomalaisten objektiivisesti mitattu fyysinen aktiivisuus, paikallaanolo ja fyysinen kunto. [The objectively measured physical activity, sedentary behavior and physical fitness of Finns]. Helsinki: Ministry of Education and Culture, Finland.

7. Koponen P, Borodulin K, Lundqvist A, Sääksjärvi K & Koskinen S. 2018. Terveys, toimintakyky ja hyvinvointi Suomessa – FinTerveys 2017 -tutkimus. [Health, functional capacity and welfare in Finland – FinHealth 2017 study]. Report 4/2018. Helsinki: Terveyden ja hyvinvoinnin laitos.

Table S1. Comparison of education, marital status and lifestyle factors between the present study sample, EsmiRs sample, ERMA sample, and the Finnish population.

	Present sample aged 51–59 (*n* = 358)	EsmiRs sample aged 51–59 (*n* = 494)	ERMA sample aged 47–55 (*n* = 1098)	Finnish women
	%/*M* ±*SD*	%/*M* ±*SD*	%/*M* ±*SD*	%/*M*
**Education %**				[1]
1. comprehensive school	0.8[b]	1.0[b]	2.2[b]	10.0
2. upper secondary school	19.5[b]	20.4[b]	21.7[b]	40.0
3. post-secondary vocational college	33.0[c]	35.4[c]	34.7[c]	22.5
4. bachelor's degree	14.5[c]	14.0[c]	12.9[c]	10.5
5. master's degree	26.3[c]	23.5[c]	23.6[c]	15.4
6. licentiate/doctoral degree	5.9[c]	5.7[c]	4.9[c]	1.6
**Marital status %**				[2]
Married	63.7	62.6	61.5	55.4
Single	7.5	7.5	8.5	22.1
Divorced	12.6	12.8	14.6	20.6
Widow	1.1	1.2	0.8	1.9
Other[a]	15.1	15.9	15.6	-
**Alcohol consumption**, l/y, M±SD	1.6 ±1.7	1.6 ±1.8	1.9 ±1.9	1.9[3]
**Risky alcohol use, %** (≥7 portions/wk)	10.1	10.5	11.3	~10[4]
**Daily smokers, %**	5.6[b]	5.9[b]	7.1[b]	13.5[5]
**BMI**, *M*±*SD*	26.6 ±3.9	25.6 ±3.9	25.5 ±3.7	28.1[5]
**Normal weight, %** (BMI <25)	52.5[a]	51.8[a]	50.7[a]	33.9[5]
**MVPA** min/d, *M* ±*SD*	47.9 ±26.7	47.8 ±27.2	49.7 ±25.9	~45[6]

[a] Includes options cohabitation, living apart together, and other. [b] Underrepresented or [c] overrepresented compared to the Finnish population sample based on chi-square test and adjusted residuals.

[1] Finnish women aged 45–59, (*n* = 521 329). Resource: Official Statistics of Finland (OSF): Educational structure of population [e-publication]. ISSN=2242-2919. Helsinki: Statistics Finland [referred: 2.10.2020]. Access method: http://www.stat.fi/til/vkour/index_en.html.

[2] Finnish women aged 47–55 (*n* = 516 155). Resource: Official Statistics of Finland (OSF): Changes in marital status [e-publication]. ISSN=1797-643X. Helsinki: Statistics Finland [referred: 2.10.2020]. Access method: http://www.stat.fi/til/ssaaty/index_en.html.

[3] The Drinking Habits Survey 2016. Finnish women aged 50–69 (*n* = 345). Resource: Härkönen J, Savolainen J, Virtala E & Mäkelä P. 2017. Suomalaisten alkoholinkäyttötavat 1968-2016. Juomatapatutkimusten tuloksia.[Results from the Finnish Drinking Habits Surveys 1968–2016]. Helsinki: Terveyden ja hyvinvoinnin laitos.

[4] The Drinking Habits Survey 2016. Finnish women aged 45–64. Resource: Lintonen T, Niemelä S & Mäkelä P. Alkoholinkäytön hälytysrajan ylittäviä käyttäjiä on Suomessa vähintään viisi prosenttia väestöstä [At least five percent of the Finnish population are high risk alcohol users. Results from the Drinking Habits Survey 2016.] Lääketieteellinen aikakauskirja Duodecim 2019;135(16):1459–66.

[5] FinHealth 2017 study, Finnish women aged 50–59 (*n* = 594 for BMI, and *n* = 650 for smoking). Resource: Koponen P, Borodulin K, Lundqvist A, Sääksjärvi K & Koskinen S. Terveys, toimintakyky ja hyvinvointi Suomessa – FinTerveys 2017 -tutkimus. [Health, functional capacity and welfare in Finland – FinHealth 2017 study]. Report 4/2018. Helsinki: Terveyden ja hyvinvoinnin laitos.

[6] Finnish women aged 40–59, *n* ~ 300. Resource: Husu P, Tokola K, Suni J, Vähä-Ypyä H, Mänttäri A, Vasankari T, et al. Suomalaisten objektiivisesti mitattu fyysinen aktiivisuus, paikallaanolo ja fyysinen kunto. [The objectively measured physical activity, sedentary behavior and physical fitness of Finns]. Helsinki: Ministry of Education and Culture, Finland; 2018.

Table S2. Comparison between the present study sample and non-participants regarding the EsmiRs baseline characteristics

	Analytic sample[a] (*n* = 358)		Non-participants[b] (*n* = 136)		Difference		
	M	*SD*	*M*	*SD*	*t*	*df*	*p*
Age	54.34	2.03	54.99	2.06	3.15	492	.002
Depressive symptoms, CES-D	.45	.36	.49	.38	1.15	491	.251
Leisure time physical activity, MET hours/wk	3.93	3.52	3.87	3.52	-.19	492	.848
Alcohol consumption, portions/wk	3.22	3.51	3.57	3.75	.96	492	.339
Extraversion	5.41	2.70	5.40	2.50	-.01	491	.992
Neuroticism	2.68	2.06	2.75	2.26	.33	491	.745
	n	%	n	%	x^2	df	p
Education					6.61	1	.010
Lower or upper secondary	191	53.4	90	66.2			
Tertiary or higher degree	167	46.6	46	33.8			
Relationship status					.03	1	.874
Living with a partner	299	74.3	102	75.0			
Not living with a partner	92	25.7	34	25.0			
Work situation					1.22	1	.270
Employed	332	92.7	122	89.7			
Not employed	26	7.3	14	10.3			
Regularity of eating					1.69	2	.430
Irregular	34	9.5	28	13.2			
Quite regular	230	64.2	81	59.6			
Regular	94	26.3	37	27.2			

[a] Participants replied to at least one COVID questionnaire.

[b] Non-participants who replied only to the EsmiRs baseline questionnaire.

Differences between groups analyzed with independent samples *t*-test or x^2-test.

Supplementary Material 2: Associations between changes in health behaviors and depressive symptoms

Table S3. Bivariate correlations between changes in health behaviors and depressive symptoms at the onset of the emergency conditions (in-pandemic-I questionnaire) (n=281)

Change variables	1.	2.	3.	4.	5.
**Perceived change**[a]					
1. Physical activity [c]					
2. Alcohol consumption [c]	-.09				
3. Eating healthier products habits [d]	-.01	.14*			
4. Eating unhealthier products [d]	-.13*	.25***	-.10		
**Calculated change**[b]					
5. Leisure-time physical activity [d]	.20***	.05	.09	.11	
6. Eating regularity [d]	.08	.01	.06	-.18**	.10

[a]Perceived change asked about in the in-pandemic–I questionnaire; bigger value represents increase. [b]Change calculated between the pre-pandemic time and in-pandemic-I questionnaire. [c] Spearman's rank order correlation coefficients presented [d]Pearson correlation coefficients presented. * $p < .05$, ** $p < .01$, *** $p < .001$.

Table S4. Bivariate correlations between changes in health behaviors and depressive symptoms at the end of the emergency conditions (in-pandemic-II questionnaire) (n=263)

Change variables	1.	2.	3.	4.	5.	6.
**Perceived change**[a]						
1. Physical activity[a]						
2. Alcohol consumption[a]	-.07					
3. Eating habits[a]	.29***	-.20*				
**Calculated change**[b]						
4. Leisure-time physical activity[b]	.32***	-.07	.07			
5. Eating regularity[b]	.03	-.10	.13*	.06		
6. Alcohol consumption[b]	-.12	.32***	-.10	-.10	-.14*	
7. Depressive symptoms[b]	-.06	.09	-.21**	-.05	-.17**	.05

[a]Perceived change asked about in the in-pandemic–II questionnaire; bigger value represents increase. Spearman's rank order correlation coefficients presented. [b]Change calculated between the pre-pandemic time and in-pandemic–II questionnaires. Pearson correlation coefficients presented. * $p < .05$, ** $p < .01$, *** $p < .001$.

Kekäläinen et al. Personality Traits and Changes in Health Behaviors and Depressive Symptoms during the COVID-19 Pandemic: A Longitudinal Analysis from Pre-Pandemic to Onset and End of the Initial Emergency Conditions in Finland. Supplementary materials.
